# High-fat diet induced discrepant peripheral and central nervous systems insulin resistance in APPswe/PS1dE9 and wild-type C57BL/6J mice

**DOI:** 10.18632/aging.202262

**Published:** 2020-12-03

**Authors:** Yujie Guo, Xiaojun Ma, Pengfei Li, Shengqi Dong, Xiaochen Huang, Xiuwen Ren, Linhong Yuan

**Affiliations:** 1 School of Public Health, Capital Medical University, Beijing 100069, P.R. China

**Keywords:** Alzheimer’s disease, cognition, high-fat diet, metabolic impairment

## Abstract

**This study was designed to examine whether AD pathological phenotype in APPswe/PS1dE9 (APP/PS1) mice exposed to continuous high-fat diet predispose these murine models to metabolic dysfunction and neuropathological impairments. One-month old male APP/PS1 and C57BL/6J mice were provided with 60% high-fat diet for 6.5 months. After dietary intervention, metabolic phenotyping, cognitive behaviors, AD-related brain pathological changes and insulin signaling were compared. high fat diet induced hyperglycemia, hypercholesterolemia, and aggravated inflammatory stress in both APP/PS1 and C57BL/6J mice. Compared with C57BL/6J control mice, APP/PS1 mice showed lower glucose transporter protein expression in liver, muscle, and brain. High-fat diet caused a decrease of glucose transporter protein expression in muscle and liver but increased cortical glucose transporter protein expression in APP/PS1 mice. High-fat diet-fed APP/PS1 mice demonstrated decreased cognitive function, as well as elevated cortical soluble amyloid-β levels and APP protein expression. Decrease in cortical IR, p-IR protein expression and p-GSK3β/GSK3β ratio were observed in high-fat diet-fed APP/PS1 mice. High-fat diet caused discrepant peripheral and central nervous system metabolic phenotype in APP/PS1 and C57BL/6J mice. AD pathological phenotype might accelerate metabolic changes and cognitive impairment in APP/PS1 mice treated with HFD.**

## INTRODUCTION

Alzheimer’s disease (AD) is a neurodegenerative disease that nowadays affects over 40 million people worldwide. With aging of population, this number is predicted to triple by 2050 [1]. However, over 95% of AD cases are sporadic with unknown etiology [2]. Due to the lack of effective treatment currently, research studies focusing on the pathophysiology, as well as the identification of potential risk factors involved in the onset of this insidious disease are mandatory.

Dysfunction of insulin signaling is a condition associated with multiple pathological states of chronic diseases, such as obesity, type 2 diabetes mellitus (T2DM) and metabolic syndrome. Studies have shown that insulin has significant effects on the brain and plays essential roles in maintaining glucose and energy homeostasis of central nervous system (CNS) [3]. This implies that defects in peripheral and brain insulin signaling as observed in the aging population may contribute to neurodegenerative disorders. Recent population-based epidemiological and clinical studies have indicated insulin resistance to be a pathogenic risk factor for the development of neurodegenerative disorders [4]. Impairments of neuronal insulin singling have been observed in AD patients, which highlights the contribution of insulin resistance to the increased risk of sporadic dementia [5]. Additionally, prediabetes experimental animal models show a phenotype of brain insulin signaling impairment [6]. Exacerbating AD-like pathology has also been found in animal models with brain insulin resistance [7]. Moreover, Jiménez-Palomares’s study further indicated that increased Aβ production prompts the onset of glucose intolerance and insulin resistance in db/+;APP/PS1 model mice [8]. Based on the evidence provided, a close relationship between insulin resistance, diabetes, and dementia seems to be established. These findings suggest that targeting the dysfunction of glucose metabolism and insulin signaling might be promising strategies to slowing down or preventing AD pathological progression.

As a mouse model of AD, APPswe/PS1dE9 (APP/PS1) is known to have typical AD-like pathologies and impaired cognition and memory. Studies assessing glucose metabolism in APP/PS1 mice have described glucose tolerance in standard diet-fed young mice [9]. Other studies that found young APP/PS1 mice fed with high-fat diet (HFD) demonstrated a phenotype of significantly impaired glucose tolerance [10]. Collectively, these data indicate a potential contribution of genetic and diet-induced insulin resistance in preceding AD pathology in aging APP/PS1 mice [11]. However, albeit the impact of high-fat diets on AD pathogenesis has been suggested [12], the main cellular targets and molecular mechanisms mediating these central insulin actions are far from being completely understood. In the present study, we focused on detecting the impacts of a continuous HFD administration on pathologies related to brain insulin signaling pathway in C57/BL6 Wild-type (C57 WT) and APPswe/PS1dE9 (APP/PS1) mice. We also attempted to evaluate whether APP/PS1 mice showed discrepant peripheral and central nervous metabolic responses to continuous HFD treatment in comparison with C57 WT mice. Our findings will reveal the deleterious effect(s) of continuously administered HFD on cognitive symptoms and provide novel insights into the role of insulin resistance in AD pathophysiology.

## RESULTS

### Effect of HFD on body weight

**Figure 1:**
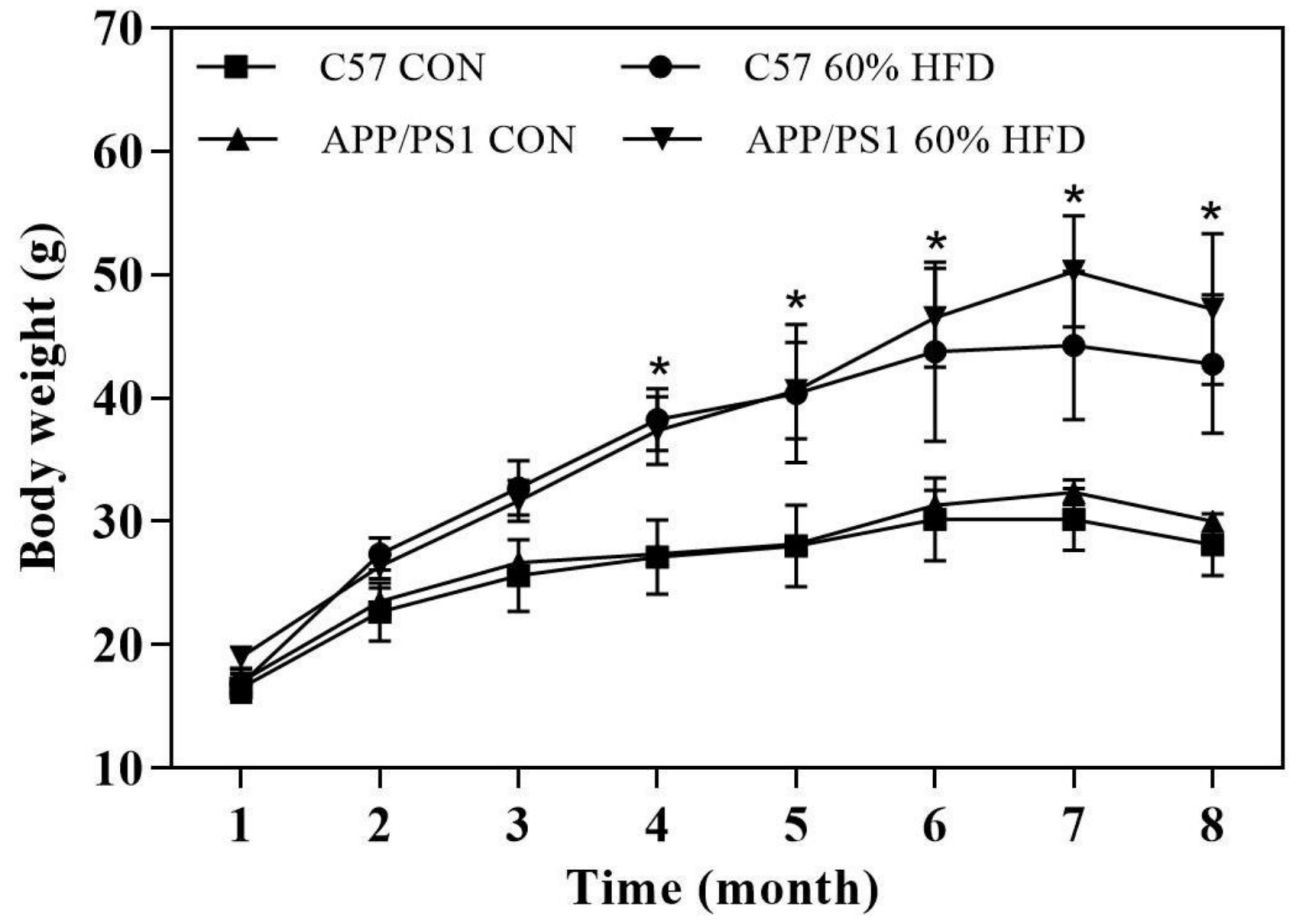
**Body weight of experimental animals during experiment *(n = 10* for each group).** C57 WT and APP/PS1 mice were fed with normal control diet or 60% high fat diet for 6.5 months and body weight were monitored. CON: control diet; HFD: high fat diet. *: comparing with control diet-treated C57 WT and APP/PS1 mice, *P* < 0.05.

After high-fat diet intervention for 6.5 months, the body weight was different across the 4 groups. As shown in Figure 1, HFD-fed C57 WT and APP/PS1 mice displayed greater weight gain than control diet-fed mice. After HFD dietary intervention for 5 months, the body weight of C57 WT mice entered a relative plateau period. In contrast, during the follow-up months, the body weight of HFD-fed APP/PS1 mice further increased and was significantly higher than HFD-fed C57 WT mice.

### Oral glucose tolerance test, serum parameters and liver histology

**Figure 2:**
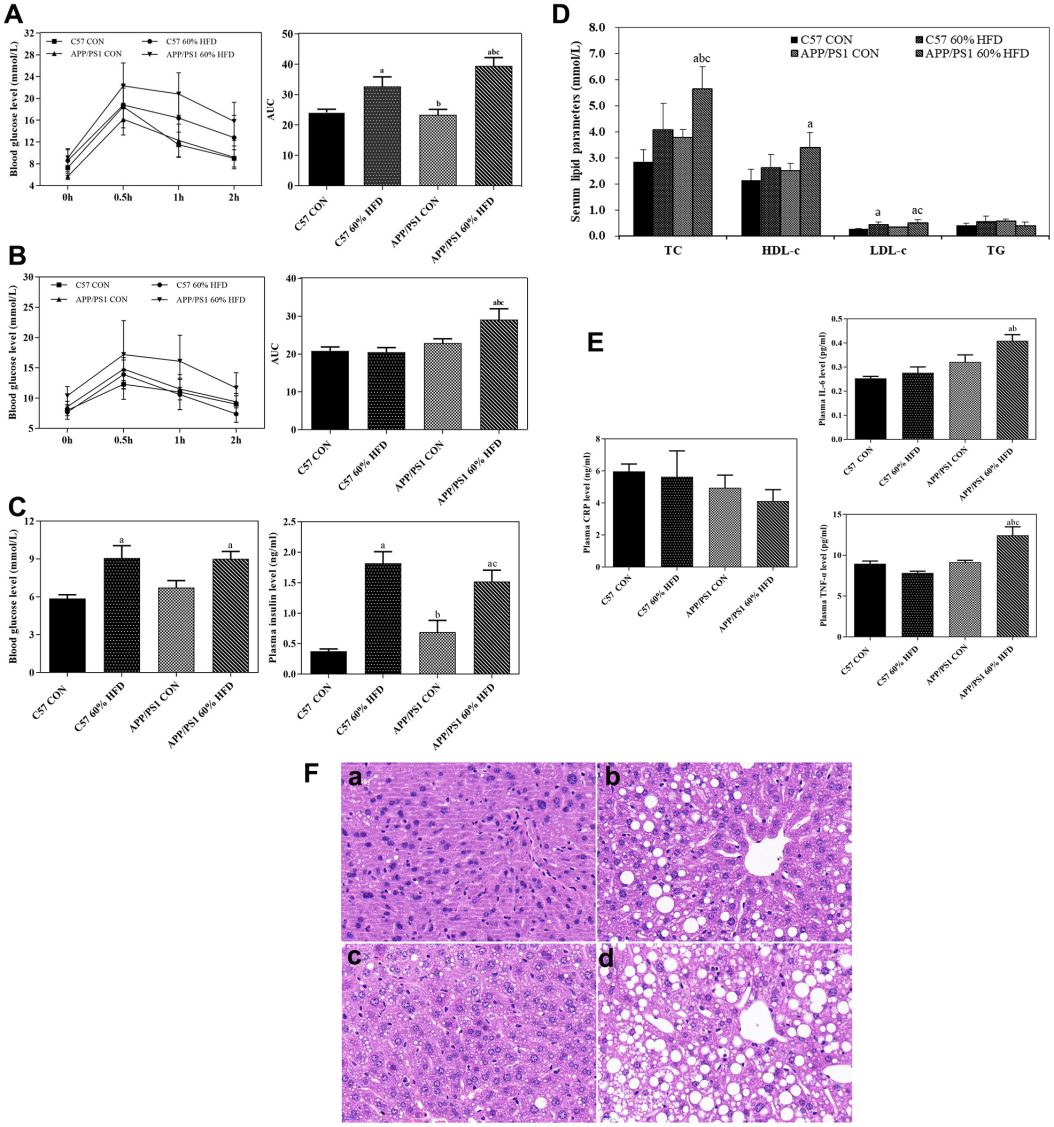
**Glucose tolerance, serum parameter levels and liver histological changes in C57 WT and APP/PS1 mice treated with different diets.** Glucose tolerance test was performed after HFD intervention for 3 (**A**) or 6.5 months (**B**) respectively. Fasting blood glucose, insulin levels (**C**), serum lipid parameters levels (**D**) and inflammatory factor levels (**E**) were measured at the end of experiment. Values presented as the mean ± SE, *n* = *10* for each group. a: comparing with C57 CON group, *P* < 0.05; b: comparing with C57 60% HFD group, *P* < 0.05; c: comparing with APP/PS1 CON group, *P* < 0.05. Liver histological changes (**F**) were detected by using HE staining method. (a) control diet-treated C57 WT mice; (b) 60% HFD-treated C57 WT mice; (c) control diet-treated APP/PS1 mice; (d) 60% HFD-treated APP/PS1 mice. Scale bar: 20μm.

The mice underwent oral glucose tolerance test (OGTT) at the age of 4 and 7.5 months. After HFD treatment for 3 months, significant change in the glycemic curve was clearly observed in HFD-treated C57 WT and APP/PS1 mice. An increase in the area under the curve (AUC) was also observed in HFD-fed C57 WT and APP/PS1 mice (Figure 2A). After HFD intervention for 6.5 months, the changes in the glycemic curve and AUC were incessantly observed in HFD-fed APP/PS1 mice, but not in C57 WT mice (Figure 2B).

As shown in Figure 2C, after 6.5-months of HFD treatment, both C57 WT and APP/PS1 mice showed significantly higher average fasting blood glucose level than normal diet-fed C57 WT and APP/PS1 mice (*P* < 0.05). In addition, HFD-fed C57 WT and APP/PS1 mice displayed significantly higher serum insulin level than normal diet-treated control mice (*P* < 0.05).

As illustrated in Figure 2D, in C57 WT mice, serum total cholesterol (TC), high-density lipoprotein cholesterol (HDL-C) and low-density lipoprotein cholesterol (LDL-C) levels presented an increased trend in response to HFD treatment compared to control mice, but no statistical significance was observed between the groups (*P* > 0.05). In APP/PS1 mice, a significant increase of serum TC and LDL-c level was observed in the HFD-fed APP/PS1 mice, and the difference between the groups reached statistical significance (*P* < 0.05).

As presented in Figure 2E, HFD intervention had no effect on serum c-reactive protein (CRP) level in C57 WT mice and APP/PS1 mice (*P* > 0.05). As compared with C57 WT control mice, APP/PS1 control mice showed higher serum interleukin-6 (IL-6) level, but no statistical significance was observed (*P* > 0.05). The level of serum IL-6 in APP/PS1 control mice was higher than that in control- or HFD-diet treated C57 WT mice. HFD significantly increased serum tumor necrosis factor-alpha (TNF-α) level in APP/PS1 mice (*P* < 0.05) but did not affect serum TNF-α level in the C57 WT mice (*P* > 0.05).

As shown in Figure 2F, HE staining results showed normal liver morphology in the C57-WT control mice, as indicated by homogeneous cytoplasmic staining and distinct cell nuclei. In contrast, the 7.5-month old APP/PS1 control mice exhibited slightly increased hepatic lipid droplets deposition. Additionally, the administration of HFD caused significant morphological changes in liver of both C57 WT and APP/PS1 mice, as demonstrated by vacuolar structures and accelerated lipid droplets deposition in liver. We also found that, in comparison with HFD-fed C57 WT mice, the HFD-fed APP/PS1 mice lost normal liver morphology and demonstrated larger number of lipid vacuoles in liver.

### Effect of HFD on behavior, brain senile plaque, Aβ content, and APP, BACE1, IDE expression

**Figure 3:**
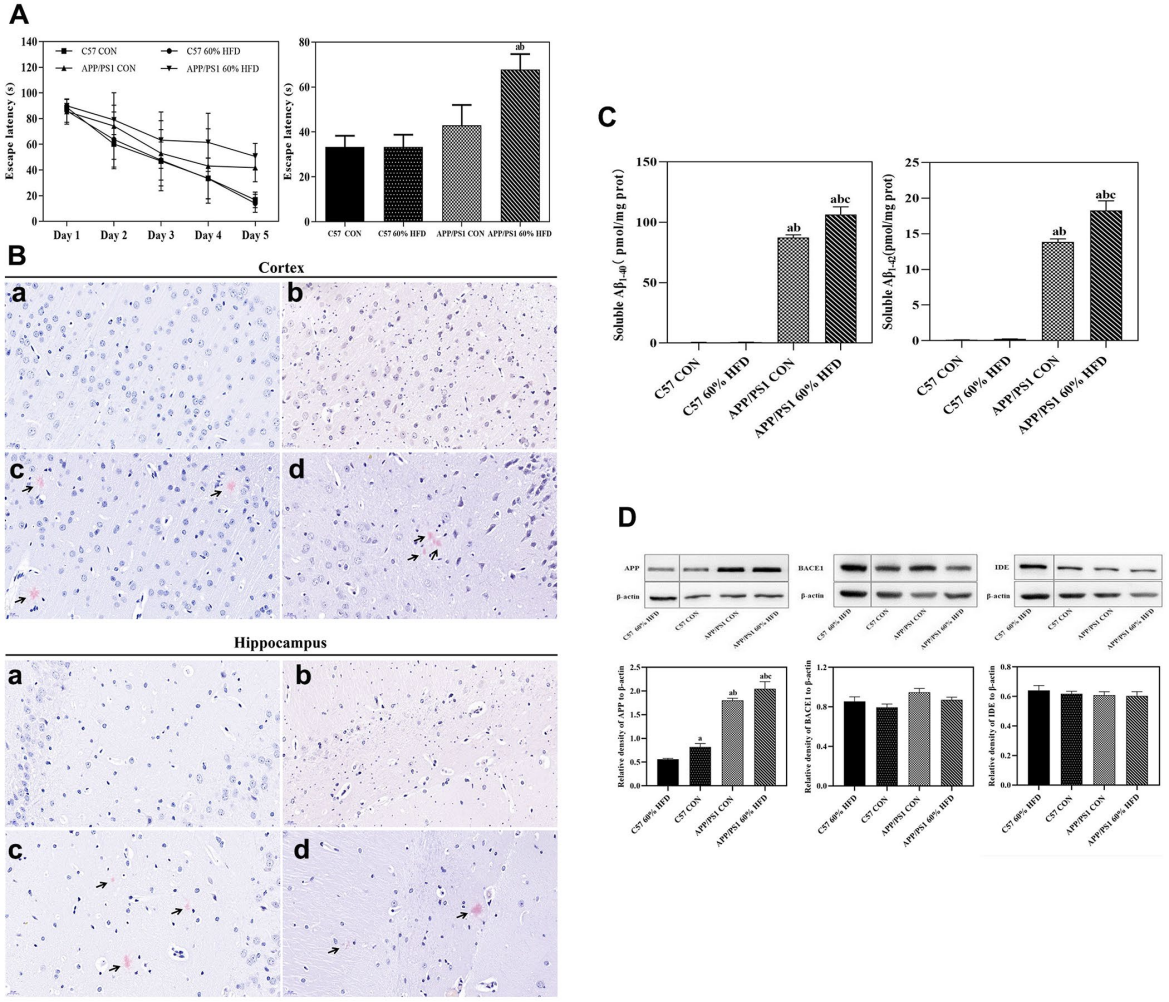
**Behavior, Aβ plaque deposit, cortical Aβ content, and APP, BACE1, IDE protein expressions in APP/PS1 and C57 WT mice treated with different diets.** APP/PS1 and C57 WT mice were fed with 60% HFD or normal control diets for 6.5 months, then, behavior was test by using MWM (*n = 10* for each group); Aβ plaque deposit was measured by using Congo red staining; cortical soluble Aβ_1-40_ and Aβ_1-42_ content were test by ELISA method; cortical APP, BACE1 and IDE protein expression was detected by Western blotting (*n = 6* at least for each group). Data were expressed as mean ± SE. (**A**) Escape latency of training and histogram of the time spent in the border area in MWM test. (**B**) Cortical and hippocampal Aβ plaque deposit. a: control diet-treated C57 WT mice; b: 60% HFD-treated C57 WT mice; c: control diet-treated APP/PS1 mice; d: 60% HFD-treated APP/PS1 mice. Scale bar: 20 μm. (**C**) Soluble Aβ_1-40_ and Aβ_1-42_ content in cortex; a: compared with C57 CON group, *P* < 0.05; b: compared with C57 60% HFD group, *P* < 0.05; c: compared with APP/PS1 CON group, *P* < 0.05. (**D**) Cortical APP, BACE1 and IDE protein expression. a: compared with C57 60% HFD group, *P* < 0.05; b: compared with C57 CON group, *P* < 0.05; c: compared with APP/PS1 CON group, *P* < 0.05.

As shown in Figure 3A, HFD treatment did not significantly affect learning ability of C57 WT mice, and HFD-fed C57 WT mice showed a similar learning profile as C57 WT control mice. As expected, the APP/PS1 mice showed inferior outcomes with the learning profile than C57 WT mice. HFD treatment showed a deleterious effect in the APP/PS1 mice, which was demonstrated by the significant increase of escape latency in APP/PS1 mice as compared to HFD-fed C57 WT mice.

As shown in Figure 3B, we did not detect senile plaques in cortex and hippocampus of the C57 WT mice. Great amounts of cortical and hippocampal senile plaques were observed in APP/PS1 control mice. The treatment of HFD had no significant effect on number and the coverage of cortical senile plaques in the APP/PS1 mice. Hippocampal senile plaques number was marginally decreased in HFD-fed APP/PS1 mice but was not significant (*P* > 0.05).

The levels of soluble Aβ_1-40_ and Aβ_1-42_ were quantified by using enzyme-linked immunosorbent assay (ELISA) method. As expected, almost no soluble Aβ_1-40_ and Aβ_1-42_ were detected in C57 WT mice (Figure 3C). APP/PS1 control mice showed higher cortical soluble Aβ contents when compared with C57 WT mice (*P* < 0.05). The level of cortical soluble Aβ_1-40_ and Aβ_1-42_ was significantly increased in HFD-fed APP/PS1 mice, and statistical significance was observed when compared to normal diet-fed APP/PS1 mice (*P* < 0.05).

As shown in Figure 3D, APP/PS1 control mice showed significant increased cortical amyloid precursor protein (APP) expression compared with C57 WT control mice (*P* < 0.05). HFD decreased cortical APP protein expression in C57 WT mice, but significantly up-regulated APP expression in APP/PS1 mice (*P* < 0.05). APP/PS1 control mice exhibited approximate β-secretase 1 (BACE1) and insulin-degrading enzyme (IDE) protein expression as C57 WT control mice. HFD treatment had no effect on cortical BACE1 and IDE protein expression in both C57 WT and APP/PS1 mice (*P* > 0.05).

### Immunohistochemical results

**Figure 4:**
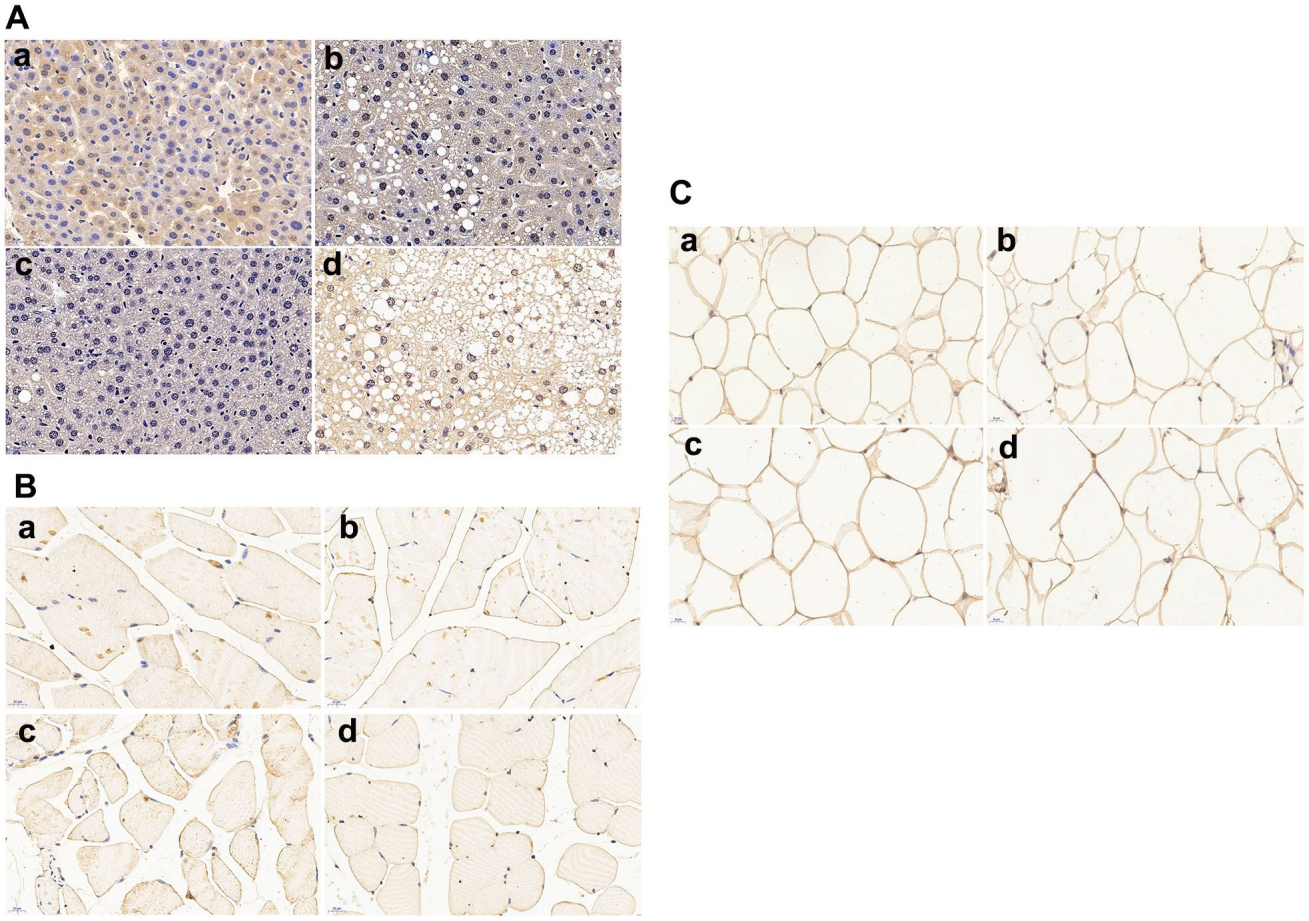
**Glucose transporters (GLUTs) protein expressions in liver, skeletal muscle and omental adipocyte (*n = 6* at least for each group).** (**A**) GLUT2 protein expression in liver tissue; (**B**) GLUT4 protein expression in muscle; (**C**) GLUT4 protein expression in omental adipocytes. a: control diet-treated C57 mice; b: 60% HFD-treated C57 mice; c: control diet-treated APP/PS1 mice; d: 60% HFD-treated APP/PS1mice.Scalebar:20μm.

As shown in Figure 4A, APP/PS1 control mice have less hepatic glucose transporter 2 (GLUT2) protein expressions than C57 WT control mice. HFD treatment caused significant lipid droplets deposition in the liver of APP/PS1 mice and further down-regulated the GLUT2 protein expression in both C57 WT and APP/PS1 mice.

Skeletal muscle GLUT4 protein expression is shown in Figure 4B. Remarkable decrease of GLUT4 expression was found in APP/PS1 control mice compared with C57 WT control mice. High fat diet significantly down-regulated the protein expression of GLUT4 in both APP/PS1 and C57 WT mice, and the lowest muscle GLUT4 protein expression was found in HFD-treated APP/PS1 mice.

Omental adipocytes GLUT4 protein expression was shown in Figure 4C. There was no significant difference of adipocyte GLUT4 protein expression between C57 WT and APP/PS1 control mice. Adipocyte GLUT4 protein expression remained unchanged in HFD-treated C57 WT and APP/PS1 mice when compared to C57 WT and APP/PS1 control mice.

### Effect of HFD on cortical GLUT3 and insulin metabolism related protein expression

**Figure 5:**
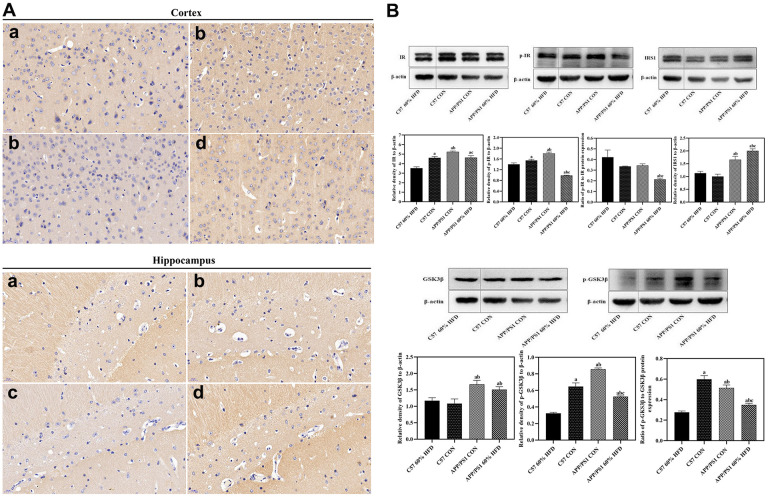
**GLUT3 and insulin metabolism related protein expression in brain of experimental animals treated with different diets (*n = 6* at least for each group).** (**A**) GLUT3 expression in cortex and hippocampus. a: control diet-treated C57 mice; b: 60% HFD-treated C57 mice; c: control diet treated APP/PS1 mice; d: 60% HFD treated APP/PS1 mice. (**B**) Cortical insulin metabolism related protein expression. a: comparing with C57 60% HFD group, *P* < 0.05; b: comparing with C57 CON group, *P* < 0.05; c: comparing with APP/PS1 CON group, *P* < 0.05.

As shown in Figure 5A, in comparison with C57 WT mice, remarkable decrease of cortical and hippocampal GLUT3 protein expressions were found in APP/PS1 control mice. HFD treatment significantly increased cortical and hippocampal GLUT3 protein expression in APP/PS1 mice but had no significant effect on HFD-fed C57 WT mice.

As shown in Figure 5B, APP/PS1 control mice showed higher cortical IR and p-IR protein expression than C57 WT control animals (*P* < 0.05). HFD significantly down-regulated cortical insulin receptor (IR) and phosphorylated insulin receptor (p-IR) protein expression in both C57 WT and APP/PS1 mice (*P* < 0.05), and the lowest cortical p-IR protein expression was observed in HFD-treated APP/PS1 mice (*P* < 0.01). High fat diet also caused dramatic decrease of p-IR/IR ratio in APP/PS1 mice (*P* < 0.05). The IRS1 protein expression was different between C57 WT and APP/PS1 control mice, and APP/PS1 control mice demonstrated significantly higher cortical IRS protein expression than C57 WT control mice (*P* < 0.05). HFD significantly induced cortical IRS1 protein expression in APP/PS1 mice (*P* < 0.05) but had no significant impact on C57 WT mice (*P* > 0.05).

APP/PS1 control mice showed higher cortical total glycogen synthase kinase-3β (GSK3β) and phosphorylated glycogen synthase kinase-3β (p-GSK3β) protein expression and p-GSK3β/GSK3β ratio than C57 WT control mice (*P* < 0.05). Cortical total GSK3β protein expression remained unchanged in HFD-fed C57 WT and APP/PS1 mice (*P* > 0.05). Down-regulation of p-GSK3β protein expression and p-GSK3β/GSK3β ratio were observed in HFD-fed C57 WT and APP/PS1 mice (*P* < 0.05), and the lowest cortical p-GSK3β protein expression was found in HFD-treated C57 WT mice (*P* < 0.05).

## DISCUSSION

Recent evidence suggests a correlation between type 2 diabetes mellitus (T2DM) and Alzheimer’s disease (AD). It has also been suggested that metabolic abnormalities as well as insulin signaling dysfunction are the common backgrounds shared by both AD and T2DM pathogenesis [13]. High-fat diet (HFD) mediated insulin resistance has been suggested to contribute to the prevalence of T2DM and AD [14, 15]. However, the impact of continuous HFD on brain and peripheral metabolic syndrome has not been well studied in experimental animals with AD pathological phenotype. In the current study, we compared the cerebral and peripheral metabolic changes between APP/PS1 and C57 WT mice in response to 6.5-month HFD treatment. Our data indicated that continuous HFD can differentially affect cerebral and peripheral insulin signaling pathways in APP/PS1 and C57 WT mice. AD pathological phenotype might predispose APP/PS1 mice to HFD-mediated metabolic syndrome.

HFD have been shown to induce hyperglycemia, hyperinsulinemia, and fatty liver disease in experimental animals [16]. To induce insulin resistance-like phenotype, we fed APP/PS1 and C57 WT mice with HFD for 6.5 months and, as expected, HFD treatment resulted in weight gain, liver histopathological changes and insulin resistance for both types of mice (Figure 1, 2). These findings are consistent with other studies which reported an increased weight gain in HFD-fed APP/PS1 mice and a lesser weight gain in control diet-fed AD model mice [17]. We also found that, after HFD treatment for 4 months, the APP/PS1 mice gained more body weight than the HFD-fed C57 WT mice (Figure 1). These findings suggest that the transgenic features and properties of the AD-model mice accelerate HFD-induced body weight gain. Furthermore, in comparison with HFD-fed C57 WT mice, HFD-fed APP/PS1 demonstrated aggregated hyperglycemia, hypercholesterolemia, hyperinsulinemia, and enhanced hepatic lipid deposition, accompanied by decreased GLUTs expression in liver and skeletal muscle (Figure 2). These results indicated that the transgenic modification predisposed APP/PS1 mice to HFD-mediated peripheral metabolic abnormalities.

Inflammation is associated with type 2 diabetes and is also apt to promote AD pathology [18]. Elevated serum inflammatory factors have been reported to contribute to peripheral insulin resistance in HFD-treated APP/PS1 mice [19]. Consistent with these reports, in the current study, we found that HFD caused remarkable elevation of serum IL-6 and TNF-α in APP/PS1 mice (Figure 2E). Moreover, HFD-fed APP/PS1 mice demonstrated a much higher serum inflammatory biomarker levels than HFD-fed C57 WT mice (Figure 2E). These data suggested that genetic modification might also increase the susceptibility of APP/PS1 mice to HFD-induced peripheral inflammation, therefore, resulting in peripheral insulin resistance.

The impact of HFD on cognition and memory of experimental animals has been a controversial element of focus. Winocur and Greenwood’s study suggested that HFD caused impairment of cognition in wild-type mice [20]; while another study reported that HFD had no effect on cognition [21]. In the present study, HFD-fed APP/PS1 mice exhibited increased escape latency and learning profile in Morris water maze (MWM) test in comparison with normal diet-treated control animals (Figure 3A), which suggests diminished cerebellar function during insulin resistance [22].

BACE1 enzyme-dependent processing of APP has been a primary source of β-amyloid (Aβ) in brain [23]. Extracellular Aβ deposition within cortex is one of the prominent pathological hallmarks of AD. The neurotoxicity, neuroinflammation, and synaptic dysfunction caused by over accumulated Aβ have been reported to play pivotal roles in AD pathophysiology and hippocampal-dependent memory decline [24, 25]. In this current study, elevated soluble Aβ1-40 and Aβ1-42 were observed in HFD-fed APP/PS1 mice (Figure 3C). Moreover, cortical APP protein expression was significantly enhanced by HFD treatment (Figure 3D). Our data show consistency with Hoet’s study, suggesting that HFD-induced insulin resistance promotes amyloidosis in brain and exacerbates cerebral AD-like pathological alterations in APP transgenic mice [26]. Overlapping properties in the metabolism of insulin and Aβ have been shown by previously reported studies [27]. Insulin-degrading enzyme (IDE) is mainly responsible for the degradation of insulin. Both insulin and Aβ are substrates of IDE, and high extracellular level of insulin may compete with Aβ for binding to IDE, therefore leading to decreased Aβ clearance and augmented Aβ levels in the brain [28]. In the present study, our results showed that the expression of cortical BACE1 and IDE protein remained unchanged in HFD-fed APP/PS1 mice (Figure 3D), indicating that the increase of cortical Aβ status was not due to the alterations in BACE1 and IDE expression. The increase of cortical APP protein expression might attribute to the significant elevation of Aβ in HFD-fed APP/PS1 mice.

Decreased glucose uptake and insulin production in the central nervous system (CNS) have been reported in aging subjects and sporadic Alzheimer’s disease patients [29, 30]. Disruptions of brain glucose utilization and metabolic hormones are key elements in AD pathophysiology [31]. In the current study, APP/PS1 control mice displayed significantly decreased cortical GLUT3 expression when compared with C57 WT mice (Figure 5A). This result was consistent with a previous study, in which glucose tolerance was reported in standard diet-fed APP/PS1 mice [32]. It is reported that AD patients demonstrate early and progressive reductions in glucose metabolism in cortical and hippocampal regions [33]. The impaired brain glucose uptake in APP/PS1 mice demonstrated that the AD pathological phenotype disturbed the insulin receptor signaling pathway and glucose metabolism in brain [34]. In agreement with these findings, elevated cortical IR, p-IR and IRS1 protein expression were also found in APP/PS1 control mice, but not in the C57 WT control mice (Figure 5B). We thus infer that the increase of brain insulin signaling pathway protein expression in APP/PS1 control mice might be a compensatory mechanism to the impaired glucose metabolism.

Significant increase of cortical GLUT3 protein expression was observed in HFD-treated APP/PS1 mice when compared with normal diet-fed control mice (Figure 5A). This outcome was consistent with previous findings, in which upregulation of GLUT3 in the cerebellum was found during experimentally induced diabetes [35]. In contrast, the HFD treatment only caused slight increase of cortical GLUT3 expression in C57 WT mice, which demonstrated an increased susceptibility of insulin signaling pathway to HFD treatment in APP/PS1 mice.

It is reported that insulin could be transported from blood across the blood-brain barrier (BBB) into the brain [36]. Exposure to high-fat diet and obesity are associated with decreased insulin transport into the mammalian brain [37]. The phosphorylation of tyrosine kinase is assumed to be the principal mechanism of the insulin receptor activation that triggers subsequent phosphorylation mediating cellular effects of insulin. Dysfunctions in the IR-mediated processes can be attributable to lowered insulin availability [38]. Our data indicates an inhibitory effect of HFD on cortical protein expression of IR, p-IR and p-IR/IR ratio, which elucidates an impaired IR-mediated signaling in brains of these HFD-fed APP/PS1 mice (Figure 5B). The mild to moderate decrease of these insulin receptor signaling related proteins in HFD-fed C57 WT mice further proved that genetic modification increased the vulnerability of these AD-model mice to HFD-induced brain insulin-related metabolic disorder. We speculate that the HFD-mediated elevation of peripheral insulin might neutralize or counteract the existed self-compensatory activation of insulin signaling in brain of APP/PS1 mice [39], and ultimately inhibit the activation of insulin receptor and its downstream targets. HFD-mediated elevated peripheral insulin could be a physiological feedback response in an attempt to facilitate insulin uptake across the BBB in these transgenic mice, since exposure to high-fat diet and obesity have been reportedly associated with decreased insulin transport into the mammalian brain [37].

Reduced phosphorylation and elevated total GSK3β protein level indicate hyper-activity of GSK3β signaling [40]. A significant increase in brain GSK3β activity may further phosphorylate and modulate the insulin receptor (IRS-1 facilitating inactivation) thus exacerbating the neurodegenerative process in diabetic APP/PS1 mice [41]. Studies have shown that GSK3β have been abnormally activated in the brains of postmortem AD samples and AD animal models, ultimately resulting in excessive Aβ formation and aggregation [42, 43]. Besides, GSK3β activation could promote tau protein hyperphosphorylation, and lead to the formation of neuro-fibrillary tangles (NFTs) and neuronal cell death [44]. In accordance with these findings, enhanced activation of GSK3β was also observed in APP/PS1 mice. Moreover, decreased p-GSK3β protein expression and p-GSK3β/GSK3β ratio were likewise found in HFD-fed APP/PS1 mice, which indicates a hyper-activation of GSK3β signaling pathway (Figure 5B). These data indicate that the AD pathological phenotype renders the APP/PS1 mice susceptible not only to impaired brain glucose uptake, but also to disturbance of CNS’s insulin signaling in response to HFD. The alterations in the activity of insulin signaling pathway components overlapping with Aβ accumulation might ultimately accelerate the development of cognitive dysfunction in HFD-fed APP/PS1 mice.

Collectively, our data suggests that high-fat diet impacts peripheral and central nervous systems insulin signaling discrepantly in APP/PS1 and C57WT mice. Genetic predisposition for developing AD might accelerate metabolic changes and cognitive impairment in APP/PS1 mice treated with HFD. These findings are consistent with epidemiological studies, suggesting that AD and T2DM often co-exist and risk-imposed to each other [45]. Targeting insulin metabolic-related metabolic manifestations might be an effective strategy to treat this currently incurable neurodegenerative disease. Although the present study addressed the association of diabetic phenotype with AD pathology, population-based neuropathological studies are necessary to uncover the pathogenic mechanisms underlying these diseases.

## MATERIALS AND METHODS

### Animals and treatment

One-month old male C57BL/6J wild-type (WT) mice and APPswe/PS1dE9 (APP/PS1) transgenic mice from experimental animal center of Capital Medical University were used in this study. According to baseline fasting blood glucose level, C57 WT and APP/PS1 mice were randomly split into control (CON) and high fat diet (HFD) groups respectively (n = 10 for each group). C57 WT and APP/PS1 control mice were treated with standard lab control diet, in which 10% kcal was derived from fat chow. HFD-treated C57 WT and APP/PS1 mice were treated with 60% high fat diet, in which 60% kcal was derived from fat. Sucrose content per gram was equivalent between the two diets. The control diet and 60% high fat diet (60% HFD) differed in the composition of fat added in the diet. All diets were purchased from SYSE Bio-tech. Co. LTD, Changzhou, China. Composition of experimental diets was listed in Supplementary Table 1. Diet intakes and body weights of animals were assessed weekly. The animals were housed under controlled room temperature and were maintained on a 12:12 light-dark cycle and allowed access to water and food ad libitum. Experiment protocols were licensed according to Capital Medical University Animal Care and Use Committee regulations and associated guidelines (AEEI-2019-071).

### Oral glucose tolerance test

Oral glucose tolerance test was performed to evaluate alterations in glucose homeostasis as previously described [46]. Briefly, all animals were fasted for 12 h, then, baseline blood glucose level was measured. Afterwards, oral gavage of glucose at a dose of 1 g/kg was undergone. Then, blood glucose levels were measured at different time points after the administration (at 30, 60 and 120 mins) by using a standard glucometer (Accu-Chek, Roche) with blood obtained from the tail tip.

### Behavioral testing

After dietary intervention, the mice were used for behavioral testing. Morris water maze (MWM) test was applied to measure the spatial learning and memory of animals according to the methodology described in previous publication [47]. Briefly, the test was performed in a white pool 100 cm in diameter filled with water tinted with nontoxic white paint and maintained at room temperature. During the acquisition phase, an escape platform (10 cm in diameter) was placed 1 cm below the water surface in one quadrant of the pool. The environment surrounding the pool was decorated with geometric objects as spatial cues. Each mouse was subjected to four trials a day for four consecutive days. Each trial began by placing the mouse randomly into a position in one of the four quadrants of the pool and allowing it to swim freely for a maximum of 60 s. After locating the platform (or being guided to the platform if the mouse failed to reach the platform after 60 s), the animal was allowed to stay on the platform for 15 s. Twenty-four hours after the acquisition phase, a 60 s probe trial was performed to determine memory retention. The escape latency (the time for animal to locate the platform in all four trials) and platform site crossings were recorded with a video tracking system (Water Maze 2.6 Institute of Materia, Chinese Academy of Medical Sciences DMS-2, Beijing, China). Results were calculated individually for each animal.

### Measurement of serum parameters

At the end of dietary intervention, all mice were euthanized. Blood samples were collected, and serums were separated using for lipid parameters measurements. Serum triglyceride (TG), total cholesterol (TC), high density lipoprotein cholesterol (HDL-C), and low density lipoprotein cholesterol (LDL-C) levels were measured using the assay kits from NanJingJianCheng Bioengineering Institute (Nanjing, China) according to the manufacturer’s instruction. The levels of serum insulin, tumor necrosis factor-α (TNF-α), interleukin-6 (IL-6) and C-reactive protein (CRP) were measured using enzyme-linked immunosorbent assay (ELISA) kits (ImmunoWay Biotechnology, USA) according to the manufacturer’s instruction. Two independent measurements were performed for each sample.

### Tissue preparation

All mice were euthanatized and sacrificed. Brain samples were removed and separated along the middle sagittal sulcus. Cortical and hippocampal regions were dissected from half of each brain and immediately stored at -80° C. The other halves were used for the histological study. Liver, skeletal muscle and omental adipose tissue samples were frozen at -80° C or fixed in formalin for biochemical assays or for histology analysis.

### Histochemical and immunohistochemical staining

The liver samples were embedded in 10% formaldehyde buffered solution, subsequently embedded in paraffin blocks, and sectioned into slices of 5 μm, and then stained with hematoxylin-eosin (HE) as previously described [48]. Specimens were examined under a light microscope.

Immunohistochemistry (IHC) assays were performed according to previous describe [49]. IHC was performed on paraffin-embedded sections using antibodies directed against GLUT2, (1:100 dilution, cell signaling technology, USA), GLUT3, (1:100 dilution, cell signaling technology, USA), and GLUT4 (1:100 dilution, proteintech, USA). Secondary DAB antibody was used for positive detection. Then, the section was observed and photographed by electric microscope (Olympus BX61).

### Measurement of brain Aβ plaque and cortical Aβ content

Cortical and hippocampal Aβ plaques were measured by using a Congo red (Key Gen Bio Tech, Nanjing, China) staining method according to the description of Oksman [50]. The levels of soluble Aβ1-40 and Aβ1-42 content in cerebral cortex were measured by using Aβ ELISA high-sensitive kits (Wako, Japan) according to the manufacturer’s instruction. Briefly, the cerebral cortex was homogenized into phosphate buffer saline (PBS) containing 0.5% SDS, 0.5% Triton X-100, and 1 mM phenylmethylsulfonyl fluoride with protease inhibitor cocktail tablets (Roche Diagnostics, Indianapolis, IN, USA). After sonication and centrifugation, the supernatant was designated as the SDS-soluble fraction. The pellet was suspended in 3 M guanidine-HCl and sonicated. Protein concentrations were determined by BCA protein assay kit (Pierce Company, Rockford, MI, USA) according to the manufacturer’s instruction. Levels of Aβ1-42 and Aβ1-40 were standardized to total protein content in the same sample and expressed as pmol of Aβ per mg protein.

### Western blotting

Tissue samples were homogenized in chilled lysis buffer (50 mM Tris-HCl pH: 7.4, 150 mM NaCl, 5 mM EDTA, 1% Triton X-100) supplemented with protease and phosphatase inhibitor cocktail. Protein concentrations of the homogenates were determined by using the BCA Protein Assay Kit (Pierce Company, Rockford, MI, USA). A total of 30 μg of protein lysis extracts were electrophoresed on Tris-tricine gradient gels, and Western blots were probed using the following primary antibodies (1:1000, Abcam, USA): APP, BACE1, IR, p-IR (Tyr1150/1151), IRS1, IDE, GSK3β and p-GSK3β (Ser9) overnight at 4° C. After washing the membrane extensively, the membrane was incubated with appropriate secondary antibodies (1:10000) for 1 h and developed using chemiluminescence reporting. Band intensities were scanned and quantified with densitometric software (ChemiDoc™ MP, Bio-Rad, USA). Results were normalized with β-actin (Cell Signaling, USA) antibodies.

### Statistical analysis

All experiments were performed in triplicate. The results were reported as mean ± SEM or mean ± SD. The data were calculated using Prism (GraphPad Software Inc., San Diego, CA, USA). Comparisons between controls and treated groups were determined by using one-way analysis of variance (ANOVA) followed by Tukey’s post hoc test. *P* < 0.05 was considered statistically significant.

## Supplementary Material

Supplementary Table 1
