# Supplementary material for: High-fat diet induced discrepant peripheral and central nervous systems insulin resistance in APPswe/PS1dE9 and wild-type C57BL/6J mice
Source: Aging (Albany NY). 2020 Dec 3;13(1):1236–50. doi: 10.18632/aging.202262 (PMC7835010; doi:10.18632/aging.202262)
Supplement: Supplementary Table 1 [file aging-13-202262-s001.pdf]

SUPPLEMENTARY TABLE

Supplementary Table 1. Composition of experimental diets (g/kg).

Nutrients in diet	Control diet	60% high fat diet
**Ingredient**		
Casein	200	200
L-Cystine	3	3
Corn Starch	315	0
Maltodextrin	35	125
Sucrose	350	68.8
Cellulose	50	50
Mineral Mix	45	45
Vitamin E	0.065	0.065
Vitamin A	0.0012	0.0012
SFA	15.48	105.38
MUFAs	17.09	112.30
PUFAs	12.45	52.51
**kcal %**		
Protein	20	20
Carbohydrate	70	20
Fat	10	60

SFA: saturated fatty acid; MUFAs: multiple unsaturated fatty acids; PUFAs: polyunsaturated fatty acids.
